# Screening for Anthrax Postexposure Antibiotic Prophylaxis—The New York City Approach

**DOI:** 10.1089/hs.2018.0025

**Published:** 2018-12-29

**Authors:** Mark Misener, David T. Starr, Allison J. Scaccia, Vibhuti Arya

**Affiliations:** Mark Misener, MD, is Field Operations Medical Specialist, the Office of Emergency Preparedness and Response, New York City Department of Health and Mental Hygiene, Long Island City, New York.; David T. Starr, MIA, is Assistant Commissioner, Bureau of Emergency Field Operations, the Office of Emergency Preparedness and Response, New York City Department of Health and Mental Hygiene, Long Island City, New York.; Allison J. Scaccia, NP, is Director, Clinical Planning Unit, the Office of Emergency Preparedness and Response, New York City Department of Health and Mental Hygiene, Long Island City, New York.; Vibhuti Arya, PharmD, is Associate Clinical Professor, College of Pharmacy and Health Sciences, St. John's University, Queens, New York.

## Abstract

Individuals exposed to spores of *B. anthracis* must take a course of antibiotics as postexposure prophylaxis (PEP) to prevent inhalation anthrax. During an anthrax event, public health authorities are responsible for conducting dispensing operations to offer PEP to exposed individuals. Jurisdictions have developed antibiotic PEP screening algorithms to determine which antibiotic is appropriate for each individual. Variability exists with regard to screening questions and dispensing decisions based on responses to those questions. It is likely that individuals with similar profiles will receive different antibiotics based solely on the jurisdiction in which they receive their PEP. This lack of consistency among jurisdictions may lead to a loss of confidence in the public health response among the public, the healthcare community, the media, and government leaders, which could compromise the response itself. We present New York City's planning assumptions, screening algorithm, a rationale for our screening questions, and our reasons for excluding screening questions asked by other jurisdictions. We hope that our efforts may assist others in developing and refining their algorithms and associated public messaging and encourage standardization with neighboring jurisdictions where appropriate.

## Abstract 2

Individuals exposed to spores of B. anthracis must take a course of antibiotics as postexposure prophylaxis (PEP) to prevent inhalation anthrax, and public health authorities are responsible for conducting dispensing operations. Jurisdictions have developed antibiotic PEP screening algorithms to determine which antibiotic is appropriate for each individual. This article presents New York City's planning assumptions, screening algorithm, a rationale for screening questions, and reasons for excluding screening questions asked by other jurisdictions.

Public health planning for and response to a large-scale release of aerosolized spores of *Bacillus anthracis*, the agent that causes inhalation anthrax, incorporates many critical elements. The identification and treatment of ill individuals, communication with the public and local stakeholders (eg, healthcare providers, media, elected officials), and the dispensing of antibiotics and vaccine as postexposure prophylaxis (PEP) to all potentially exposed individuals must be considered.

Depending on the location, quantity, and mechanism of an anthrax release, thousands to millions of people from many jurisdictions may be exposed and require PEP. These individuals will need to obtain PEP from points of dispensing (PODs) or other dispensing options operated under the jurisdiction of state and local health authorities.

State and local public health authorities throughout the United States, including the New York City Department of Health and Mental Hygiene (DOHMH), have developed screening algorithms to determine what type of antibiotic PEP is dispensed to clients in response to an anthrax attack. Yet, as has been described, these were developed using general guidance, but not specific recommendations, from federal health authorities.^1^ Therefore, variation exists among these algorithms with regard to screening questions and the dispensing choices based on responses to those questions. Given this variation, it is highly likely that clients with similar profiles will receive different antibiotics based solely on the jurisdiction in which they receive their PEP. While this fact may not adversely affect the desired outcome of prevention of inhalation anthrax, such inconsistency is likely to erode trust in the public health response among the public, the healthcare community, the media, and government officials and lead to confusion and perceptions of inequity, both of which could negatively affect compliance with public health recommendations and adherence to the full PEP regimen.

Each jurisdiction must, when developing its algorithm, incorporate evidence-based principles (eg, which antibiotics are most effective and appropriate), inventory concerns (eg, which antibiotics will be readily available for immediate dispensing), and operational considerations (eg, how can a large number of individuals receive the appropriate antibiotic in a rapid, efficient manner). In this article, we present New York City's anthrax antibiotic screening algorithm for a deliberate anthrax release, discuss the rationale for our choice of screening questions, including our rationale for excluding some common questions, and describe how we intend to handle clients who require additional medical evaluation. This article does not address screening or other considerations related to the administration of anthrax vaccine.

## Background

The Centers for Disease Control and Prevention (CDC) recommends that all individuals exposed to spores of *B. anthracis* immediately begin a 60-day course of an oral antibiotic and a 3-dose course of anthrax vaccine for PEP. Doxycycline and ciprofloxacin are FDA approved as equivalent first-line antibiotics for PEP as they are equally effective and have similar susceptibility profiles among naturally occurring *B. anthracis* isolates.^2,3^ Clindamycin or amoxicillin is recommended for individuals who have an allergy or other contraindication to both doxycycline and ciprofloxacin, although neither is FDA approved for anthrax PEP.^2,4^ Jurisdictions rely on the CDC's Strategic National Stockpile (SNS) for the antibiotics necessary for large-scale PEP operations.^5^

The DOHMH is assigned the core competency of mass prophylaxis/vaccination in the New York Citywide Incident Management System (CIMS) and is therefore authorized to direct such operations in New York City.^6^ The DOHMH relies primarily on a neighborhood-based POD network to dispense PEP. While the vast majority of staff in NYC PODs are nonclinical, there are clinical staff at each POD in the medical evaluation unit.

## Assumptions

In the development of our plan, we have assumed that:

1. New York City will receive doxycycline and ciprofloxacin from the SNS in quantities that are sufficient for large-scale dispensing at PODs;
2. The strain of *B. anthracis* will be susceptible to both of these first-line antibiotics;
3. We may receive quantities of ciprofloxacin suspension and amoxicillin from the SNS, but these quantities will be insufficient for allocation across a large network of POD sites to ensure availability at all sites given variable rates of consumption;
4. Antibody-based options for anthrax PEP (eg, raxibacumab) are not considered suitable options for consideration at PODs as they are administered by infusion in a healthcare setting;
5. Clients who must take ciprofloxacin suspension, amoxicillin, or clindamycin can be directed from a POD to a local pharmacy; and
6. The number of clients directed to a local pharmacy should be minimized:
  a. Supplies of ciprofloxacin suspension, amoxicillin, and clindamycin in the commercial supply chain are limited; and
  b. Clients will experience delay in beginning their PEP as opposed to those who receive antibiotics in a POD.

## Screening Questions and Algorithm

A screening form with the following questions must be completed for each individual receiving antibiotics for anthrax PEP. The DOHMH has a designee pickup policy that allows a client to present completed screening forms for him- or herself and up to 5 other individuals at a POD. Clients are provided with an explanation of each question to assist them in answering the question correctly. [Note: These questions and explanations have been translated into the 12 most commonly spoken languages in NYC and are available upon request.]

1. Has the person who will take the antibiotic ever had a severe allergic reaction to doxycycline or tetracycline antibiotics that required medical attention?
2. Is the person who will take the antibiotic pregnant?
3. Has the person who will take the antibiotic ever had a severe allergic reaction to ciprofloxacin (Cipro) or other fluoroquinolones that required medical attention?
4. Does the person who will take the antibiotic have myasthenia gravis?
5. Is the person who will take the antibiotic taking tizanidine (Zanaflex^®^)?
6. Is the person who will take the antibiotic taking theophylline?
7. Does the person who will take the antibiotic have kidney disease or is he/she on dialysis?
8. Is the person who will take the antibiotic unable to swallow pills?
9. Does the person who will take the antibiotic weigh less than 76 pounds or 35 kilograms?

**Figure 1.**
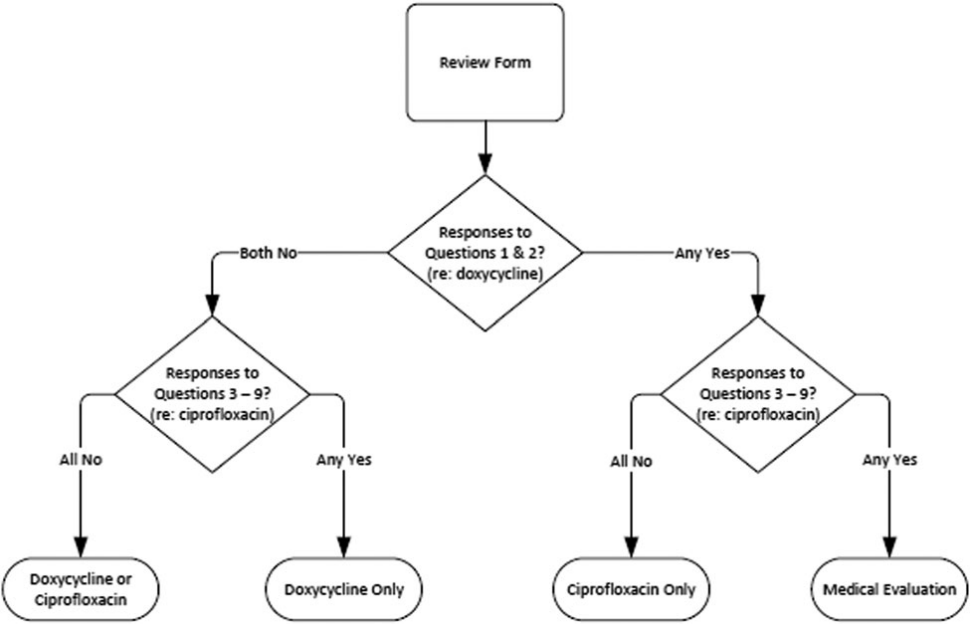
NYC DOHMH Screening Algorithm for Anthrax Antibiotic Postexposure Prophylaxis

Clients will be screened for the most appropriate antibiotic PEP based on their answers to these 9 questions using the following algorithm (Figure 1).

1. If the client answers “No” to all questions 1-9, then the client will receive doxycycline, but may receive ciprofloxacin in the event that supplies of doxycycline are low.
2. If the client answers “No” to questions 1 AND 2 and “Yes” to ANY of questions 3-9, then the client has an allergy or other contraindication to ciprofloxacin, or can't swallow pills, and will receive only doxycycline.
3. If the client answers “Yes” to question 1 OR 2 and “No” to ALL of questions 3-9, then the client has an allergy to doxycycline or is pregnant and will receive only ciprofloxacin.
4. If the client answers “Yes” to question 1 OR 2 and “Yes” to ANY of questions 3-9, then the client has an allergy to doxycycline or is pregnant AND has an allergy or other contraindication to ciprofloxacin, or can't swallow pills, and will be directed to medical evaluation.

## Rationale for Screening Questions

Based on our assumptions, our choice of screening questions directs the vast majority of clients to receive either doxycycline or ciprofloxacin. We acknowledge that for some clients, neither doxycycline nor ciprofloxacin would be preferred because of a potential for adverse events based on individual medical history. However, we chose screening questions that prioritize prevention of inhalation anthrax, which is likely to cause mortality or severe morbidity, over the avoidance of possible non–life-threatening adverse drug reactions.

The rationale for each screening question we include and the effect of its answer on the algorithm is described below.

1. *Has the person who will take the antibiotic ever had a severe allergic reaction to doxycycline or tetracycline antibiotics that required medical attention?*

Doxycycline is contraindicated in individuals who have shown hypersensitivity to any of the tetracyclines.^7^ The DOHMH will not dispense doxycycline to clients who answer “Yes” to this question.

2. *Is the person who will take the antibiotic pregnant?*

The Food and Drug Administration (FDA) has listed doxycycline and ciprofloxacin as Pregnancy Categories D and C, respectively,^7,8^ and doxycycline is not typically prescribed for use in pregnant women based on reports of potential risks associated with tetracycline. Current CDC anthrax PEP recommendations include prioritizing ciprofloxacin over doxycycline in pregnant women when ciprofloxacin is available.^2^ However, existing literature suggests that adverse events experienced by pregnant women who take tetracycline are not similarly experienced by those who take doxycycline.^4,9-11^

The DOHMH will not dispense doxycycline to clients who answer “Yes” to this question unless they also answer “Yes” to any of questions 3 through 9. These clients will be referred to medical evaluation, where they will receive doxycycline or a prescription for an alternative antibiotic if they answer “Yes” to question 1.

3. *Has the person who will take the antibiotic ever had a severe allergic reaction to ciprofloxacin (Cipro) or other fluoroquinolones that required medical attention?*

Ciprofloxacin is contraindicated in individuals who have shown hypersensitivity to any of the fluoroquinolones.^8^ The DOHMH will not dispense ciprofloxacin to clients who answer “Yes” to this question.

4. *Does the person who will take the antibiotic have myasthenia gravis?*

Myasthenia gravis is a neuromuscular disease that is characterized by fluctuating muscle weakness and fatigue. The FDA has issued a black box warning that ciprofloxacin may exacerbate muscle weakness in people with a history of myasthenia gravis.^8^

While myasthenia gravis is relatively uncommon and has an estimated prevalence of ∼20 per 100,000 people in the United States,^12^ serious morbidity (including dysarthria, dysphagia, difficulty chewing, and severe weakness or paralysis of oropharyngeal and respiratory muscles) or mortality may occur in people with exacerbation of disease.

The DOHMH will not dispense ciprofloxacin to clients who answer “Yes” to this question.

5. *Is the person who will take the antibiotic taking tizanidine (Zanaflex^®^)?*

Tizanidine is a short-acting muscle relaxer used to treat spasticity caused by multiple sclerosis, amyotrophic lateral sclerosis, spastic diplegia, back pain, and some injuries to the spine and/or central nervous system. It is sometimes prescribed for other conditions, including migraine headaches, sleep problems, and fibromyalgia. It is metabolized mainly in the liver, and ciprofloxacin interferes with this metabolism, which may result in greatly elevated plasma concentrations of tizanidine. This may result in increased lethargy, somnolence, confusion, depressed cardiac function, respiratory depression, and coma. Because of the potential risks for severe outcomes, individuals who take tizanidine should not concomitantly take ciprofloxacin.^13^ Finally, individuals who have been receiving at least 20 mg of tizanidine for at least 9 weeks and who may wish to change to another medication must taper off of tizanidine slowly to avoid the risk of serious withdrawal adverse reactions.

The DOHMH will not dispense ciprofloxacin to clients who answer “Yes” to this question.

6. *Is the person who will take the antibiotic taking theophylline?*

Theophylline is a medication used to treat symptoms and reversible airway obstruction associated with chronic asthma and other chronic lung diseases, including emphysema and chronic bronchitis. It is metabolized mainly in the liver, and ciprofloxacin interferes with this metabolism, which may result in greatly elevated plasma concentrations of theophylline. This may increase the risk of seizure and/or cardiac dysrhythmia. Because of the potential risks for life-threatening outcomes, individuals who take theophylline should not concomitantly take ciprofloxacin if another antibiotic is appropriate and available.^8^

The DOHMH will not dispense ciprofloxacin to clients who answer “Yes” to this question unless they also answer “Yes” to question 1. These clients will be referred to medical evaluation, where they will receive ciprofloxacin unless they answer “Yes” to any other of questions 3 through 9. They will be advised to contact their healthcare provider within 24 hours to have their theophylline level monitored.

7. *Does the person who will take the antibiotic have kidney disease or is he/she on dialysis?*

Ciprofloxacin is eliminated primarily by renal excretion. However, it is also metabolized and partially cleared through the biliary system of the liver and through the intestine. While these alternative pathways appear to compensate for the reduced renal excretion in individuals with renal impairment, some modification of dosage may be recommended depending on the severity of renal dysfunction.^8^ Renal toxicity has been reported in some cases of individuals with acute overdosage of ciprofloxacin.

POD clinical staff will not have the means to assess the severity of kidney disease and make appropriate dose adjustments of ciprofloxacin for individuals who report this condition.

DOHMH will not dispense ciprofloxacin to clients who answer “Yes” to this question unless they also answer “Yes” to question 1 (severe allergy to doxycycline). These clients will be referred to medical evaluation, where they will receive ciprofloxacin (if they have not indicated they have another contraindication to ciprofloxacin), be advised to take their first dose, and then to immediately contact their dialysis center, nephrologist, or other healthcare provider to determine whether they should adjust further doses of ciprofloxacin.

8. *Is the person who will take the antibiotic unable to swallow pills?*

Individuals who are unable to swallow pills must take a liquid form of either doxycycline or ciprofloxacin. Doxycycline can be crushed and mixed with food or drink, and DOHMH will make crushing instructions available to those who need them. The FDA has not issued crushing instructions for ciprofloxacin because of concerns that the extreme unpalatability of crushed ciprofloxacin will adversely affect adherence.

DOHMH will not dispense ciprofloxacin to clients who answer “Yes” to this question unless they also answer “Yes” to question 1 (severe allergy to doxycycline). For those clients, medical evaluation staff will provide a prescription for ciprofloxacin suspension.

9. *Does the person who will take the antibiotic weigh less than 76 pounds or 35 kilograms?*

Individuals who meet this criterion and who can take doxycycline should take less than the adult dose.^7^ Doxycycline may be crushed and mixed with food or drink to ensure that the client receives the appropriate dose. Instructions on crushing doxycycline will be made available to POD clients.

DOHMH will not dispense ciprofloxacin to clients who answer “Yes” to this question, as it may not be crushed (see question 8 above), unless they also answer “Yes” to question 1 (severe allergy to doxycycline). For those clients, DOHMH will dispense ciprofloxacin to those who weigh between 67 and 76 pounds and have answered “No” to question 8 (unable to swallow pills). Medical evaluation staff will provide a prescription for ciprofloxacin suspension to those weighing less than 67 pounds.^8^

We have reviewed screening forms and algorithms from other health departments, including examples developed by other large cities with demographics similar to New York City as well as those from smaller jurisdictions. During our review, we noted that some questions are standard on all forms (eg, previous allergic reaction), but there is variability regarding other questions (eg, breastfeeding). We opted to exclude a number of questions that are asked by some other jurisdictions. As discussed previously, under ideal circumstances, some clients should not take either doxycycline or ciprofloxacin. However, these antibiotics will be immediately available in a POD, and, due to the emergent nature of an anthrax emergency, the likelihood of a severe outcome from inhalation anthrax, and the delay clients will experience obtaining an alternative antibiotic from a local pharmacy, we have chosen not to include questions regarding non–life-threatening adverse drug reactions to either antibiotic. In an attempt to further reduce risk from PEP-related adverse events, our public messaging will encourage clients who meet various criteria (eg, breastfeeding, seizure disorder/epilepsy, history of tendon rupture, chemotherapy, anticoagulant use) to consult with their physician once they begin their PEP. Our rationale for not including these screening questions is described below.

1. *Questions regarding seizure disorder/epilepsy*

Doxycycline and ciprofloxacin, as well as antibiotics from other classes, may lower the level of various anti-seizure medications.^7,8^ This effect may increase the risk of seizure in individuals with seizure disorder, including epilepsy. Ciprofloxacin-associated seizures have been described in patients with certain risk factors, including advanced age and renal insufficiency.^14^

Neither doxycycline nor ciprofloxacin are without risk in these individuals, and medical evaluation staff will lack the means to more fully evaluate individuals with seizure disorders.

Screening for this condition is unlikely to influence the choice of antibiotic in a POD setting.

2. *Questions regarding a history of tendon rupture*

The FDA requires a black box warning that ciprofloxacin has been associated with an increased risk of tendinitis and tendon rupture.^8^ This adverse reaction most frequently involves the Achilles tendon but may also involve the rotator cuff, hand, biceps, thumb, and other tendons. The risk of these adverse reactions is increased in individuals over 60 years of age, in patients taking corticosteroids, and in people with kidney, heart, and lung transplants. However, a history of tendon rupture is not considered a contraindication for ciprofloxacin.

Screening for this non–life-threatening condition may lead to an increase in individuals who require a prescription for an alternative antibiotic.

3. *Questions regarding breastfeeding*

Both doxycycline and ciprofloxacin are excreted in human milk.^7,8^ The amount of either absorbed by the nursing infant is unknown. There is a potential for serious adverse reactions in infants nursing from mothers taking either of these antibiotics. Neither antibiotic has been shown to create more risk than the other.

Screening for breastfeeding is unlikely to influence the choice of antibiotic in a POD setting.

4. *Questions regarding chemotherapy agents*

Interactions between both doxycycline and ciprofloxacin with methotrexate have been reported. There is a potential for an interaction by both antibiotics with other chemotherapeutic agents.^7,8^

Screening for use of chemotherapy agents is unlikely to influence the choice of antibiotic in a POD setting.

5. *Questions regarding anticoagulant drugs*

Both doxycycline and ciprofloxacin can affect an individual's response to anticoagulant drugs,^7,8^ and individuals taking anticoagulants concomitantly with one of these antibiotics should monitor their prothrombin time and INR accordingly.

Screening for use of anticoagulant drugs is unlikely to influence the choice of antibiotic in a POD setting.

## Medical Evaluation of Clients Reporting Contraindications

A client directed to medical evaluation will consult with a licensed physician, nurse practitioner, or other clinician who has authority to prescribe antibiotics. The clinician will review the client's answers to all 9 questions, with particular attention to any “Yes” response, in order to determine the validity of the client's answers. As an example, a client may indicate that he or she has had a serious allergic reaction to doxycycline, which, after evaluation by a clinician, is not actually supported by the person's history.

Medical evaluation staff will follow a standardized procedure to conduct their review and will determine whether a client should receive doxycycline or ciprofloxacin at the POD, or ciprofloxacin suspension or an alternative antibiotic recommended by CDC for use as anthrax PEP. Medical evaluation staff will provide a prescription for ciprofloxacin suspension or an alternative antibiotic for the small number of clients who require these options and direct them to a retail pharmacy.

## Conclusion

We have presented our planning assumptions, anthrax antibiotic screening algorithm, our rationale for our choice of screening questions, and our medical evaluation function. The screening algorithm has been integrated into current NYC DOHMH policy and is regularly used in our POD leadership training.

We hope that this article will assist responders in preparing for and responding to an anthrax emergency and that they will consider standardizing their screening algorithms with neighboring jurisdictions whenever possible. We understand that some jurisdictions may not operate under the same assumptions as NYC (eg, capacity to allocate ciprofloxacin suspension and amoxicillin across a POD network). However, a thorough consideration of these possible variables in advance of an anthrax emergency will enable local planners to incorporate those assumptions into their plans and be better prepared to explain differences, if necessary, at the time of a response with clear, aligned messaging.

We strongly believe that a consistent, evidence-based antibiotic screening approach will maximize public trust, promote efficiency at PODs, and increase the likelihood of a successful response to a large-scale anthrax event.
